# The influence of coughing on cerebrospinal fluid pressure in an *in vitro *syringomyelia model with spinal subarachnoid space stenosis

**DOI:** 10.1186/1743-8454-6-17

**Published:** 2009-12-31

**Authors:** Bryn A Martin, Francis Loth

**Affiliations:** 1 École Polytechnique Fédérale de Lausanne, Integrative Bioscience Institute, Laboratory of Hemodynamics and Cardiovascular Technology, Lausanne, Switzerland; 2 Departments of Mechanical and Biomedical Engineering, University of Akron, Akron, OH, USA

## Abstract

**Background:**

The influence of coughing, on the biomechanical environment in the spinal subarachnoid space (SAS) in the presence of a cerebrospinal fluid flow stenosis, is thought to be an important etiological factor in craniospinal disorders, including syringomyelia (SM), Chiari I malformation, and hydrocephalus. The aim of this study was to investigate SAS and syrinx pressures during simulated coughing using *in vitro *models and to provide information for the understanding of the craniospinal fluid system dynamics to help develop better computational models.

**Methods:**

Four *in vitro *models were constructed to be simplified representations of: 1) non-communicating SM with spinal SAS stenosis; 2) non-communicating SM due to spinal SAS stenosis with a distensible spinal column; 3) non-communicating SM post surgical removal of a spinal SAS stenosis; and 4) a spinal SAS stenosis due to spinal trauma. All of the models had a flexible spinal cord. To simulate coughing conditions, an abrupt CSF pressure pulse (~ 5 ms) was imposed at the caudal end of the spinal SAS by a computer-controlled pump. Pressure measurements were obtained at 4 cm intervals along the spinal SAS and syrinx using catheter tip transducers.

**Results:**

Pressure measurements during a simulated cough, showed that removal of the stenosis was a key factor in reducing pressure gradients in the spinal SAS. The presence of a stenosis resulted in a caudocranial pressure drop in the SAS, whereas pressure within the syrinx cavity varied little caudocranially. A stenosis in the SAS caused the syrinx to balloon outward at the rostral end and be compressed at the caudal end. A >90% SAS stenosis did not result in a significant Venturi effect. Increasing compliance of the spinal column reduced forces acting on the spinal cord. The presence of a syrinx in the cord when there was a stenosis in the SAS, reduced pressure forces in the SAS. Longitudinal pressure dissociation acted to suck fluid and tissue caudocranially in the SAS with a stenosis.

**Conclusions:**

Pressures in the spinal SAS during a simulated cough *in vitro *had similar peak, transmural, and longitudinal pressures to *in vivo *measurements reported in the literature. The pressure wave velocities and pressure gradients during coughing (longitudinal pressure dissociation and transmural pressure) were impacted by alterations in geometry, compliance, and the presence of a syrinx and/or stenosis.

## Background

The influence of coughing on the hydrodynamic environment in the spinal subarachnoid space (SAS) is complex and not fully understood. Coughing has been shown to result in large and abrupt cerebrospinal fluid (CSF) pressure fluctuations which arise by communication between the CSF and intrathoracic pressures, through the venous system [1,2]. In a healthy person, the abrupt CSF pulsation is absorbed without causing tissue damage [3]. It has been postulated that abnormal distribution or absorption of the pressure pulse, resulting from pathological CSF system geometry or compliance, can cause damage to the neural tissues and pain for the patient [1,4]. In many cases, abnormal CSF system geometry or compliance can be attributed to a flow blockage (stenosis) in the spinal SAS (arachnoiditis) or at the foramen magnum (Chiari I malformation), both of which can be associated with syringomyelia (SM), a neuropathology characterized by development of a fluid-filled cavity or syrinx within the spinal cord (SC). If the cavity does not communicate with the SAS directly (as in a cyst), the condition is a non-communicating SM. Thus, an investigation of the influence of CSF blockage, the presence of a syrinx, and SAS compliance on cough pressure pulse distribution and absorption is needed.

Abrupt maneuvers influencing CSF pressure such as coughing, Valsalva maneuver, and sudden postural changes are thought to be an important factor influencing SC cyst pathogenesis. Williams provided a substantial body of work investigating the importance of longitudinal pressure dissociation (LPD) in the SAS during coughing and Valsalva maneuver [1-10]. Häckel *et al*. also measured LPD in patients with Chiari malformation [11]. Williams proposed a "suck" and "slosh" theory for syrinx formation and expansion, respectively. These theories were primarily based on *in vivo *measurements of craniospinal LPD produced by abrupt maneuvers and prolonged by CSF flow obstruction. The maximum craniospinal LPD in healthy persons during coughing and Valsalva was found to be ~ 35 and 5 mmHg, respectively, and LPD persisted for 1 to 2 s after coughing [3]. LPD was also found in patients with CSF flow blockage during coughing to be in excess of 100 mmHg, persisting for more than 10 s [3,6,9]. The results indicated that SAS flow blockage elevated and prolonged LPD and Williams contended that LPD was an important factor in assessment of CSF system function and influenced syrinx pathogenesis [6].

Greitz postulated that the Venturi effect could play an important role in syrinx formation and distension in SM [12]. The Venturi effect is characterized by a localized drop in fluid pressure when fluid moves through a narrowed pathway and can be applied to steady incompressible laminar flows when viscous and other losses are negligible. Greitz hypothesized that a Venturi effect in the spinal SAS with CSF stenosis produces "relatively low CSF pressure in the narrowed CSF pathway" causing "a suction effect on the spinal cord that distends the cord during each systole" [12].

It has been documented that pain in SM can be exacerbated by coughing, sneezing, straining, and sitting [13-17]. Bertrand noted in 1973 that "there is no doubt that in these three cases, coughing, straining, and postural changes caused hydrodynamic phenomena which dramatically influenced the symptoms produced by pre-existing SC cyst and also modified the size and extent of these cysts." [18]. Hall *et al*. studied animals with an induced syrinx and obtained findings that indirectly supported "the possibility that transmission of thoracic pressures to the spinal SAS with compression of the syrinx is a principle force that enlarges the syrinx." [19]. Changes in intra-abdominal pressure during baby delivery may also be associated with a change in syrinx size [20]. Oldfield *et al*. postulated that a ball-valve effect, occurring at the foramen magnum during coughing or other maneuvers, could be a primary mechanism for syrinx formation and distension [21]. In 2003, Sansur *et al*. observed that peak SAS pressures were higher during coughing in SM patients with headache (~ 71 mmHg) than in patients without headache (~ 49 mmHg), and that after surgical intervention these pressures decreased to ~ 41 and 45 mmHg, respectively [22]. Berciano *et al*. also provided a study examining the relation of coughing and pain in SM patients [16].

A number of computational studies have been conducted examining the influence of a coughing-type pressure or flow pulse to the CSF system with SM [23-28]. The work of Carpenter *et al*. proposed that an "elastic jump" produced during coughing could provide one mechanism for syrinx distension [25,29]. However, Elliott *et al*. have since shown that an elastic jump is not likely [30]. Bertram *et al*. have carefully examined the transmission of cough-like pressure pulses in a computational model of SM and found that the spinal CSF pressure wave speed was influenced by the material properties of the cord tissue and dura mater, fat, and bone [24]. In another model, Bertram *et al*. found that focal spinal arachnoiditis produced significant tensile radial stress on the SC resulting in transiently lower pressure within the SC which could help explain movement of fluid into the syrinx cavity [28]. Bilston *et al*. used computational models to examine the influence of SAS stenosis permeability [26] and importance of arterial driven CSF flow through the extracellular spaces during normal cardiac driven CSF pulsations [31,32]. Cirovic provided a detailed analytical analysis of wave propagation within the fluid filled coaxial elastic tube system present in SM and concluded that the syrinx may be characterized by abnormally slow CSF pulse propagation speed [23]. These computational studies have provided theoretical insights and important pressure information, but employed simplifications to the *in vivo *system and would benefit from additional comparison between *in vivo *and *in vitro *measurements.

Although cough pressure measurements have been acquired *in vivo*, they are scarce and lack the spatial and temporal detail required for precise understanding of the influence of CSF system compliance and SAS stenosis during coughing. In particular, the pressure measurements by Williams were taken at two longitudinal locations along the spinal SAS, and typically did not include measurement of pressure inside the syrinx [1-10,33-35]. The paucity in SAS pressure measurements during coughing *in vivo *is invariably due to the invasive means required to obtain them.

The complex spinal SAS pressure environment during a cough is not well understood. A better understanding of the pressure gradients (LPD and transmural pressure, TP) within the SAS during coughing and how they are impacted by alterations in geometry, compliance, and presence of a syrinx and/or stenosis may help understand the pathogenesis of SM. Thus, in the present study temporal and spatial pressure distribution are examined through the use of *in vitro *models representative of various conditions associated with SAS flow obstruction. *In vitro *experiments of this type are hydrodynamically similar to that observed in a patient and have provided detailed information about the relationship of pressure, flow, and SC motion [36]. Previous *in vitro *experiments examined the SAS pressure distribution during normal CSF flow pulsations [37]. These experiments found that the interaction of the syrinx and stenosis resulted in significant LPD and TP as well as significant syrinx wall motion that led to a diastolic valve mechanism and rostral tensioning of the spinal cord. In the present study we investigated the pressure distribution in a similar series of *in vitro *models subjected to a cough-type flow impulse. Close attention was given to pressure gradient trends which result in mechanical distension of the SC, since tissue distension is likely related to damage.

## Methods

### Experimental models

**Figure 1:**
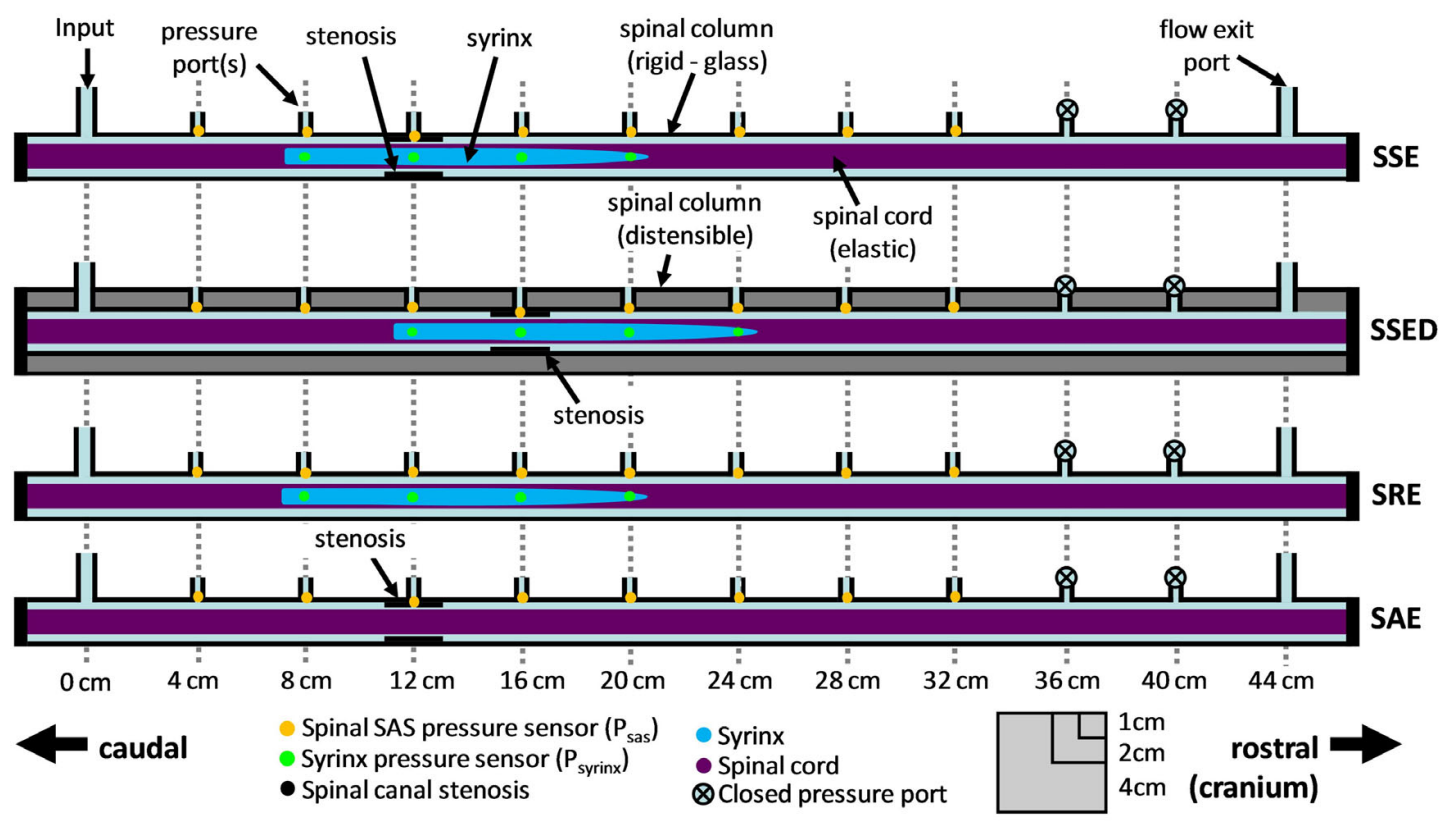
**Schematic diagram of the *in vitro *models for coughing experiments indicating the location of the stenosis, syrinx, and flow input**. Ports for pressure sensors were sited at 4 cm intervals in the subarachnoid space and in the syrinx. The simulated cough pressure pulse was inserted at the input (caudal end). SSE = stenosis and syrinx model, SSED = stenosis and syrinx model with distensible spinal column, SRE = stenosis removed model, and SAE = stenosis alone model.

Four experimental models representative of various conditions associated with spinal SAS stenosis were constructed, with or without a syrinx, with a flow input port, and pressure sensors in the syrinx and SAS (Figure 1). The spinal stenosis with syrinx model (SSE) was constructed to be representative of a non-communicating SM patient with a SAS stenosis located near the midsection of the syrinx cavity, as may occur in post-traumatic SM. The stenosis removed model (SRE) was constructed having an identical non-communicating syrinx to the SSE, but without a spinal stenosis. The third model (SAE), had a spinal stenosis but without a syrinx. Table 1 provides a summary of geometric and mechanical parameters for each model.

**Table 1:** Summary of model parameters.

Variable	Dimension	Description
D_SAS_	15.6 ± 0.3 mm	diameter of subarachnoid space

D_SC_	10.0 ± 0.2 mm	diameter of spinal cord

T	1.2 ± 0.1 mm	thickness of the glass tube used to form the spinal column

T_SSED_	12.0 ± 0.5 mm	distensible tube thickness for SSED

D_syrinx_	7.0 ± 0.2 mm	diameter of syrinx (constant until 28.7 mm from rostral end of syrinx, tapered to 3.2 mm after that point)

L_SC_	480 ± 2 mm	length of spinal cord and spinal column

L_syrinx_	132 ± 1 mm	length of syrinx

L_stenosis_	20 ± 1 mm	length of spinal subarachnoid space stenosis

%_stenosis_	>90%	percent of spinal subarachnoid space blocked by stenosis

ID_stenosis_	10.7 mm	approximate inner diameter of stenosis

E_SC_	SSE = 0.32 MPa SRE = 0.83 MPa SAE = 0.52 MPa SSED = 0.52 MPa	Young's modulus of spinal cord

E_SSED_	SSED = 1.99 MPa	Young's modulus of distensible spinal column (SSED)

Key: Tolerances are estimated from the manufacturing process tolerances and supplier specifications. SSE = stenosis with syrinx model, SSED = stenosis with syrinx model with distensible spinal column, SRE = stenosis removed model, and SAE = stenosis alone model.

The SC of the SSE, SRE, and SAE models was constructed with an isotropic linearly elastic polymer (Sylgard 184, Dow Corning, Midland, MI, USA) with a Young's modulus of 0.32, 0.83, and 0.52 MPa, respectively (Table 1). The two-part polymer was thoroughly mixed with a 20:1 base to hardener ratio, degassed with a vacuum pump (EW-07531-40, Cole-Parmer, Vernon Hills, IL, USA) in a chamber (5305-1212, Nalgene Labware, Rochester, NY, USA) for 2 h, and injected into a custom designed aluminium mold with a cavity diameter and length of 10 and 480 mm, respectively. A custom manufactured aluminium cylinder, with a diameter and length of 7 and 132 mm, respectively, was positioned within the SC mold by a centering pin to form the syrinx. The cylinder diameter was tapered from 7 down to 3.2 mm starting 28.7 mm from the rostral end of the syrinx (Figure 1). The SC and centering pin were carefully removed from the mold with soapy water after curing for ~ 48 hours. Only one SC injection mold was constructed. Thus, each SC was cast separately resulting in a variation in SC Young's modulus (Table 1). The SC Young's modulus was determined by conducting uniaxial stress-strain measurements on three cylindrical shaped polymer specimens obtained from each SC mixture during the casting process. A rigid glass spinal column was custom manufactured for each model (SSE, SRE, SAE) with an inner diameter, thickness, and length of 15.6, 1.2, 480 mm, respectively. Ten SAS pressure ports spaced at 4 cm intervals were present in each spinal column (Figure 1). Syrinx and spinal cord dimensions were based on *in vivo *measurements from a Chiari I malformation patient with SM [36]. Additional construction and apparatus details for SSE, SRE, and SAE are provided in Martin *et al*. [37].

In models with a stenosis, a ~ 2 cm length annular shaped stenosis was constructed with rubber tubing (part # 14-150-2F, Fischer Scientific, Rochester, NY, USA) and fitted into the spinal SAS blocking >90% of the total area (10.7 and 15.6 mm inner and outer diameter, respectively). A small hole was punched radially through the stenosis to enable pressure recording through the adjacent pressure port (Figure 1). In addition, a narrow 2 mm diameter channel through the centre of the SC caudal to the syrinx cavity was present (not indicated in figures). This channel was used to guide the catheter transducers into the syrinx and was blocked during the experiments.

**Figure 2:**
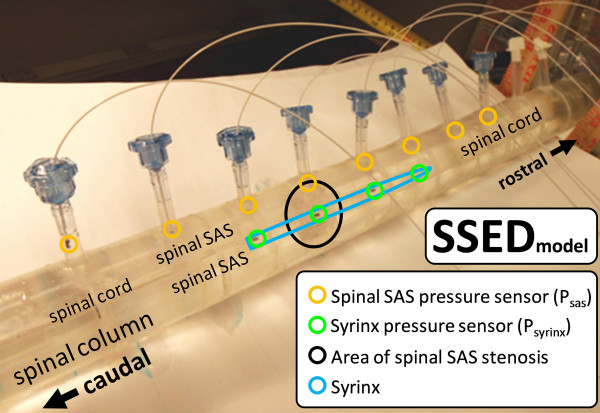
**Photograph of the syrinx and stenosis model with distensible spinal column (SSED)**. Approximate location of the syrinx, SAS stenosis, spinal cord, and spinal column are indicated. The small vertical tubes connecting to the larger tube are ports used for SAS pressure measurements. Wires extending from the ports are the catheters used for pressure measurement in the SAS (also located in syrinx cavity).

A fourth model was constructed to reproduce the SSE model but with the addition of a distensible spinal column (SSED, Figures 1, 2). SSED was identical to SSE with the exception that the spinal column was formed by an elastic polymer having an inner diameter, thickness, and Young's modulus of 15.6 mm, 12 mm, and 1.99 MPa, respectively (Table 1, Figure 2). The spinal cord for SSED had a Young's modulus of 0.52 MPa and syrinx dimensions identical to SSE and SRE (Table 1 and Figure 1). The distensible spinal column was constructed by mixing the elastic polymer with a 10:1 base-to-hardener ratio and casting it between two concentric pipes, the larger having an inner diameter of 39.6 mm and the smaller with an outer diameter of 15.6 mm (Table 1). After the polymer cured for 48 h, it was carefully removed from the pipes with soapy water and cut to the desired length of 480 mm (Figure 2).

Water was chosen as the fluid to occupy the SAS and syrinx in all of the experiments due to its similarity in density and viscosity to CSF [38]. Piezoelectric pressure catheters (Millar Instruments SPR-524, 3.5F, 100 cm, Houston, USA) were positioned at 4 cm intervals along each of the models (Figure 1 and 2). In SSE, SRE, and SSED, four sensors were also located in the syrinx cavity at 4 cm-spaced locations (Figure 1 and 2). Note, in the SSED, the syrinx and stenosis were both located 4 cm rostral to the other models (Figure 1).

### Cough simulation

In order to simulate a cough in the spinal SAS, each model was connected to a computer controlled pulsatile pump which produced an abrupt 5 ms pressure pulse through a tube connected to the input port located at the caudal end (Figure 1). The caudal end was chosen for cough input because *in vivo *the CSF cough pressure pulse moves in the caudocranial direction [1,25]. The cough-like pressure pulse travelled from the pump into the model through a 7.6 m long rigid tube with a 6.35 mm inner diameter (SPEB25 polyethylene, Watts, North Andover, USA). The tubing caused a significant delay between the production of the pulse at the pump and the arrival of the pulse at the flow input port. The pressure distribution within the spinal SAS caused by the cough pulse was reported relative to its arrival time at the model. Additional details on the sensor calibration routine and experimental setup is provided in Martin *et al*. [37].

### Pressure measurements and data handling

The sensor voltage was amplified (model 880-0129, Millar Instruments), acquired via a DAQ card (USB-6229, National Instruments, Austin, TX, USA), recorded in Labview, and processed using MATLAB (R2006b, ver. 7.3, The MathWorks, Inc., Natick, USA). Data was collected for a total of five cough trials on each model at 17 kHz resolution for a total of 4000 samples (0.235 s) from each pressure sensor. Each trial was compared and found to produce nearly identical sensor response. Thus, sensor data from only one of the trials on each model was used for further analysis. Pressure measurements from each sensor *P*(*t*), in the SAS and syrinx at predetermined axial coordinates were displayed as the pressure *P*_*r*_(*t*), relative to the baseline pressures *P*_*b *_at each sensor, calculated by:

The baseline pressure in the SAS and syrinx was calculated by averaging the first 340 samples of data for each sensor before the cough pressure pulse arrived at the sensors. Initial pressure in the SAS for all experiments was ~ 15 mmHg. Initial pressures in the syrinx varied and for SSE, SRE, and SSED were 14.1, 5.5, and 28.4 mmHg, respectively. Wide variations in the relative pressure between the syrinx and SAS have been measured *in vivo *[19,39-41].

Transmural pressure (TP) across the syrinx wall was obtained by subtracting pressure sensor signals in the syrinx from adjacent external pressures in the SAS (Figure 1) as in:

Longitudinal pressure dissociation (LPD) was calculated by subtracting the cervical from the lumbar SAS pressure located at positions 32 and 4 cm, respectively:

The cough pulse pressure was obtained by subtracting the minimum from the maximum pressure during cough.

SAS and syrinx pulse pressures gave an estimate of the magnitude of force fluctuation acting equally in all directions (fluid pressure). The pulse pressure of the TP measured the force normal to the SC surface causing expansion or contraction of the syrinx cavity in the radial direction. LPD measured the longitudinal force acting on the CSF system to suck or push CSF or tissue in the caudocranial or craniocaudal direction during the cough pulse.

Pressure wave propagation speed in the spinal SAS was computed by dividing the distance between sensors by the time delay for the arrival of the foot of the pressure waveform at each sensor. Arrival time of the pressure wave foot was detected by calculating the maximum pressure gradient with respect to time at each sensor (max[*dP*(*t*)/*dt*]). The slope from a linear least square fit of the distance versus arrival time data for the series of sensors in the SAS and syrinx was used to quantify the pressure wave propagation speed [42]. Probability for each linear fit was computed using Microsoft Excel 2003 regression analysis toolbox (Microsoft, Redmond, USA). *P*-values less than 0.05 were considered to be statistically significant. The wave speed may not be constant over the entire region of interest and thus, a linear fit only gives an approximation of wave speed. This may be more evident *in vivo *when geometry or tissue properties vary along the spinal SAS.

## Results

### Pressure changes over time

**Figure 3:**
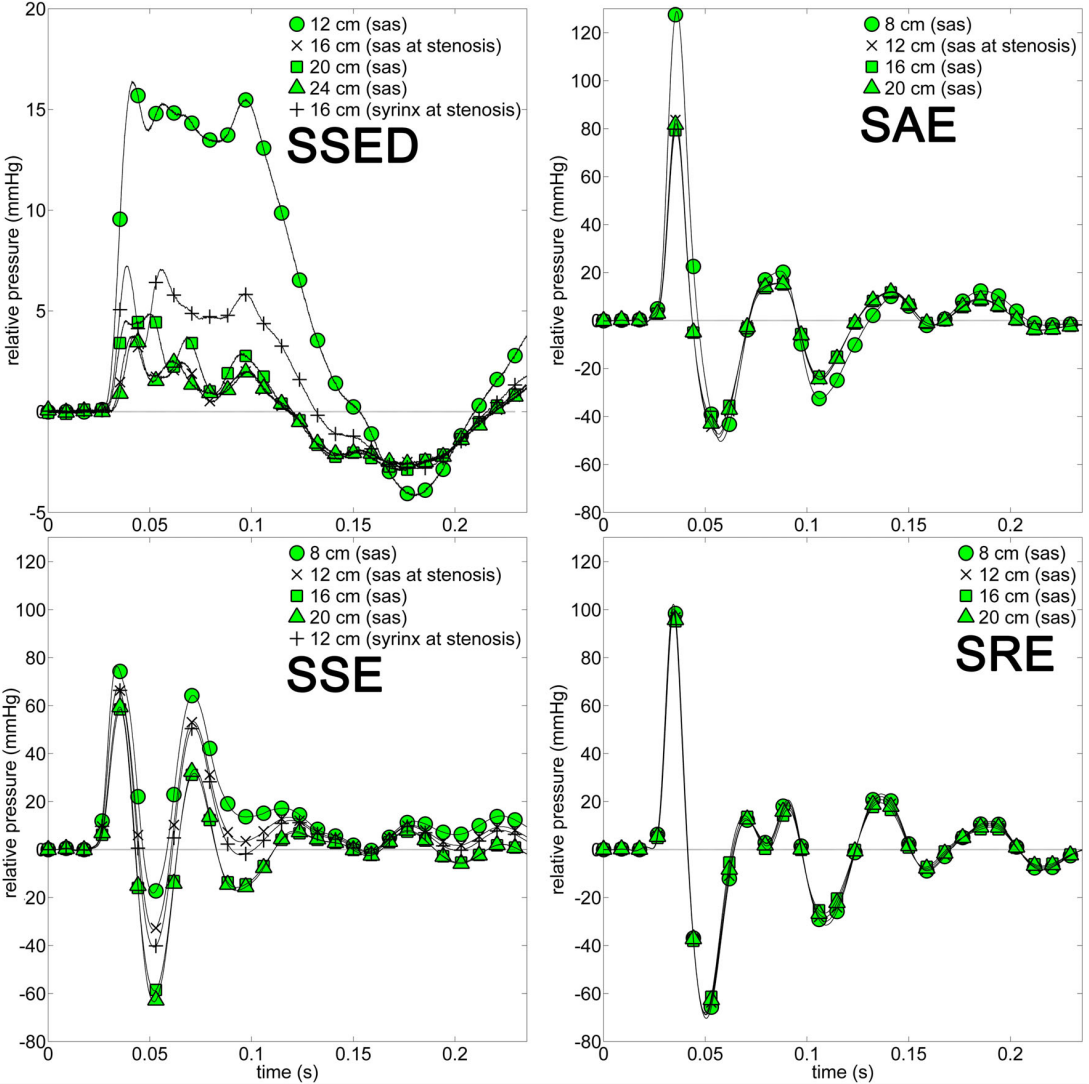
**Pressures relative to baseline (*P*_*r*_(*t*)) plotted over time for various axial locations for each experimental model**. Symbols identify location of pressure sensors. Sampling frequency was 17 kHz. Note: SSED has a different pressure scale to the other plots. Compliance in the spinal column (SSED) resulted in a pressure waveform that exhibited greater damping and less oscillatory response than in the other situations. SSED, SAE, SSE, and SRE as for Figure 1.

For each model, the pressure fluctuation created in the SAS and syrinx by a simulated cough was generally characterized by a spike in pressure followed by oscillations that damped out after ~ 200 ms. The magnitude of the maximum pressure varied widely between the models. Figure 3 shows the pressure variations over time for each model at different axial locations in the SAS and syrinx. In the distensible spinal column model (SSED), the pressure waveform exhibited greater damping and less oscillatory response than in the rigid models (SAE, SSE, SRE, Figure 3). The complete pressure data sets recorded in each model during the coughing experiments are provided in the Additional File 1.

**Figure 4:**
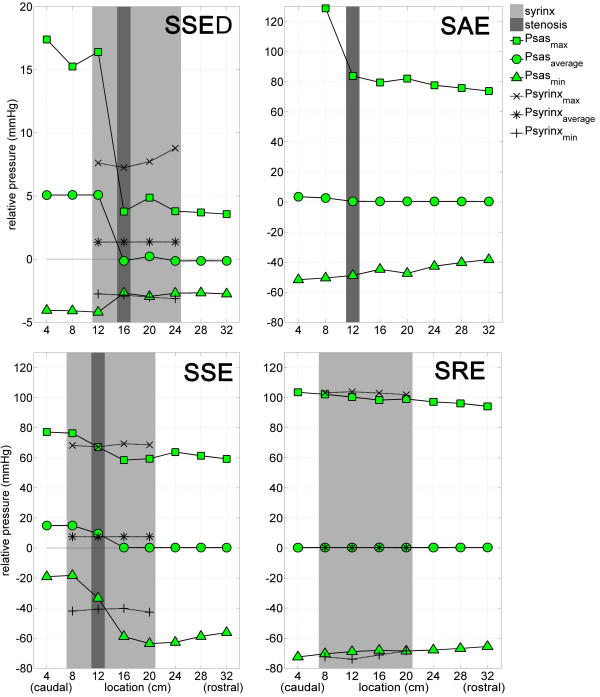
**Maximum, average, and minimum pressures (*P*_*r*_(*t*)) relative to baseline in the SAS and syrinx plotted against location for each experimental model during simulated cough**. The cough pressure pulse was applied caudally at 0.0 cm on the x-axis and travelled caudorostrally through the spinal SAS. The light grey rectangle denotes location of the syrinx cavity (when present), the dark grey vertical stripe denotes the location of the SAS stenosis. Note: SSED has a different pressure scale to SSE, SRE, and SAE. SSED, SAE, SSE, and SRE as for Figure 1.

Plotting the longitudinal distribution of maximum, average, and minimum pressures allows comparison between the complex pressure profiles of the four experimental models (Figure 4). In SSE, SAE, and SSED, maximum SAS pressure decreased moving caudocranially across the stenosis, while in SRE pressure was nearly constant along the spinal SAS. The greatest pressure drop across the stenosis was observed in SSED (as a percentage of the original pressure fluctuation). Relatively small pressure changes occurred rostral to the stenosis. Peak pressures reached in SSED, SAE, SSE, and SRE were 17, 140, 77, and 103 mmHg, respectively and maximum pulse pressures in SSED, SAE, SSE, and SRE, were 21, 192,127, and 176 mmHg, respectively (Figure 3).

Interestingly, SAS pulse pressure in SSE increased caudocranially across the stenosis from 95 to 123 mmHg. After the cough pressure pulse passed the stenosis, the average SAS pressure did not vary substantially from the initial SAS pressure, being nearly zero for all models (Figure 4). The minimum SAS pressure moving caudocranially across the stenosis increased slightly in SSED and decreased in SSE.

### Longitudinal pressure dissociation (LPD)

**Figure 5:**
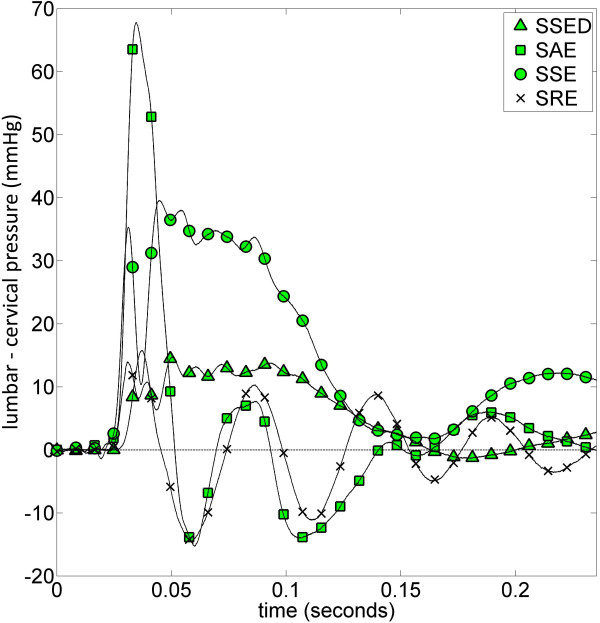
**Longitudinal pressure dissociation (LPD) (lumbar - cervical) measured in the SAS for each experimental model during coughing: *LPD*(*t*) = *P*_*SAS*_,_4*cm*_(*t*) - *P*_*SAS*_,_32*cm*_(*t*) plotted against time**. A positive deflection in LPD indicates that a pressure gradient is available to suck fluid and/or tissue towards the cranium. Peak LPD sucks fluid and/or tissue toward the cranium in SSED, SSE, and SAE, while in SRE LPD oscillates back and forth around zero. Sampling frequency for each experiment was 17 kHz. SSED, SAE, SSE, and SRE as for Figure 1.

SAS LPD (lumbar - cervical) during cough varied significantly between experimental models (Figure 5). Peak, pulse, and average LPD in the SAS are tabulated in Table 2. SAE had the greatest LPD peak and pulse. The minimum LPD of all four models was -14.5 mmHg in SRE. Average LPD during coughing (Table 2) was greatest with a rigid model having a stenosis and syrinx (SSE) and least when the stenosis was removed (SRE). Flexibility of the spinal column reduced the average LPD in SSED in comparison to SSE. While SRE did have nearly zero average LPD, it had significant peak and pulse LPD of 14 and 28 mmHg, respectively. Closer inspection of the LPD pressure trace for SRE indicates that the pressure fluctuations oscillated about zero or equilibrium (Figure 5). This was different in the SSE, SSED, and SAE models where there was non-zero average LPD (Table 2).

**Table 2:** Peak, pulse, and average longitudinal pressure dissociation in the subarachnoid space between caudal and rostral sensors (SSE, SSED, SRE and SAE as for Table 1).

	Longitudinal pressure dissociation, LPD (mmHg) *LPD*(*t*) = *P*_*SAS*_,_4*cm*_(*t*) - *P*_*SAS*_,_32*cm*_(*t*)		
**model**	**peak**	**pulse**	**average**

SSED	15.8	17.2	5.2
SSE	35.6	40.2	14.7
SAE	67.8	83.1	3.2
SRE	14.0	28.5	-0.1

Key: Tolerances are estimated from the manufacturing process tolerances and supplier specifications. SSE = stenosis with syrinx model, SSED = stenosis with syrinx model with distensible spinal column, SRE = stenosis removed model, and SAE = stenosis alone model.

### Transmural pressure (TP)

Average syrinx pressure showed small or no variation along the entire length of the syrinx in SSE, SRE, and SSED (Figure 4) and closely followed the SAS pressure at the stenosis (Figure 3). The TP force which would cause compression or ballooning of the syrinx is the difference between average syrinx and average SAS pressure in Figure 4. This indicates that the presence of a stenosis in SSE and SSED caused the syrinx to be compressed caudal to the stenosis and expanded rostral to the stenosis during coughing. When the stenosis was removed (SRE), the difference in average pressure in the SAS and syrinx was nearly zero (Figure 4).

**Figure 6:**
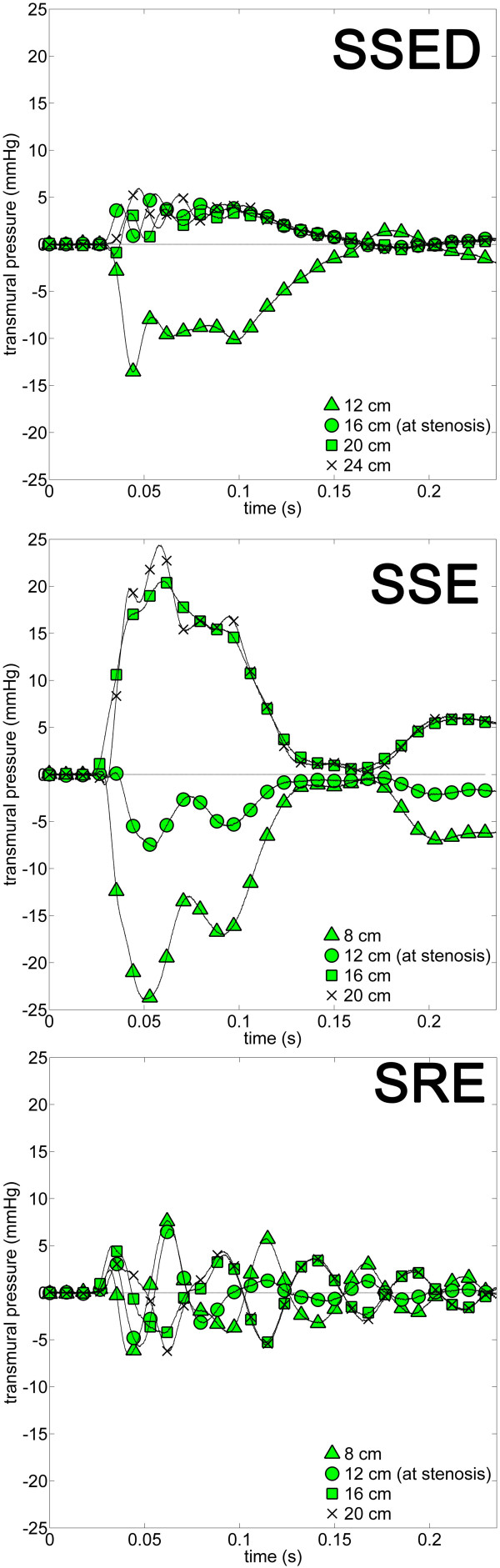
**Transmural pressure (TP) measured at various locations along the syrinx in each model (SSED, SSE, and SRE) during coughing: *TP*(*t*) = *P*_*r*, *syrinx*_(*t*) - *P*_*r*_,_*SAS*_(*t*) and plotted against time**. Positive pressure trace indicates that pressure in the syrinx is greater than the SAS at adjacent sensor locations. Negative pressure trace indicates that syrinx pressure is less than the SAS. Legend symbols identify pressure sensor location. The presence of a SAS stenosis (SSED and SSE) could cause the syrinx to balloon outward at its rostral end and be compressed at the caudal end. When the stenosis was removed (SRE), TPs were smaller and oscillated about zero. SSED, SAE, SSE, and SRE as for Figure 1.

TP during the cough varied significantly for SSE, SRE, and SSED (Figure 6). In all models with a syrinx (SSE, SRE, and SSED) the TP phase inverted about the midsection of the syrinx cavity. In SRE, the TP oscillated between syrinx compression on one side and ballooning on the other, and vice versa. On average, greater TPs were observed in SSE than in SRE. SSED had smaller TPs than the analogous rigid model (SSE).

### Pressure wave propagation speed

**Figure 7:**
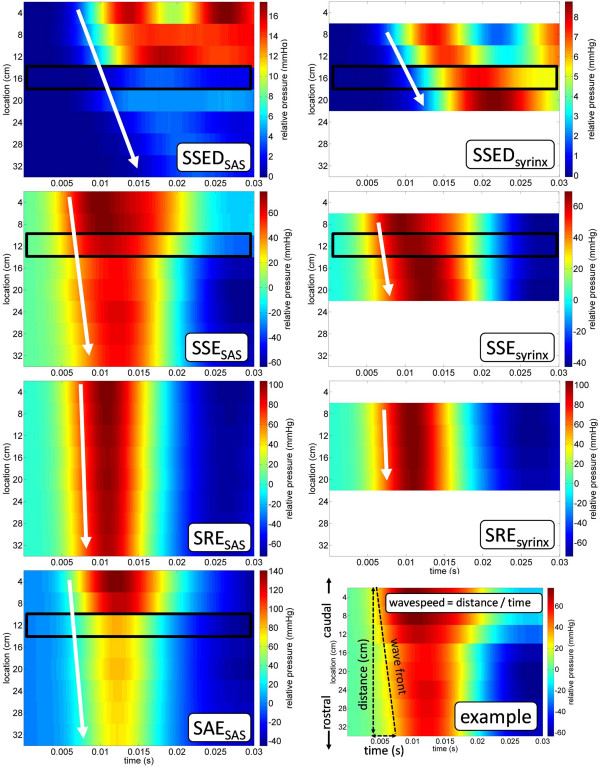
**Spatial versus temporal pressure distribution for each model in the SAS and syrinx**. Each black box indicates a pressure measurement obtained from a sensor located at a stenosis. Slope of the white arrows indicates the direction and approximate speed of the pressure wave propagation. The example (bottom right) provides a visual interpretation of the wave speed calculation technique. Note: full coughing pressure data set has been truncated temporally to visualize the foot of pressure wave arrival. SSED, SAE, SSE, and SRE as for Figure 1.

Wave propagation speeds in the SAS and syrinx varied widely between models, both temporally and spatially (Figure 7). Each black box indicates a pressure measurement obtained from a sensor located at a stenosis. Slope of the white arrows indicates the direction and approximate speed of the pressure wave propagation. These plots are in a similar form to those calculated numerically by Bertram *et al*. [24]. A summary of the wave propagation speed and maximum slope of SAS pressure rise results is provided in Table 3.

**Table 3:** CSF pressure wave speed in the spinal SAS/syrinx and maximum rate of SAS pressure rise.

Model	SAS wave speed (m/s) [*p*-value]	Syrinx wave speed (m/s) [*p*-value]	Maximum rate of SAS pressure rise (mmHg/s)
SSED	23.6 [0.0004]	24.9 [0.004]	1,700
SSE	155 [0.0001]	118 [0.026]	8,000
SAE	169 [0.0008]	N/A	13,200
SRE	399 [0.0009]	680 [0.051]	10,600

Wave speed and pressure rise were significantly smaller in the distensible model (SSED) than in the rigid models (SSE, SAE, and SRE). SSE, SSED, SRE and SAE as for Table 1.

The beginning of the pressure wave front for all models can be observed starting at time 0.005 s (Figure 7). The propagation of the wave front moves down the spinal column, in the direction of the white arrow, arriving at the rostral end (32 cm) producing a negative slope. The wave speed in the SAS and syrinx was found to be about six times slower in the flexible model (SSED) than the rigid models (Table 3). The greatest wave speed was calculated to be present in SRE (399 and 680 m/s in the SAS and syrinx, respectively), where no flow obstruction was present. The stenosis reduced the wave speed by a factor of two in SSE and SAE compared to SRE. Wave speed in the syrinx was found to be slightly slower than in the SAS for SSE, and when the stenosis was removed (SRE) wave speed was found to be greater in the syrinx than in the SAS. The maximum slope of the pressure rise at onset of the cough pulse was estimated to be 1,700 and 13,200 mmHg/s in SSED and SAE, respectively (Table 3).

## Discussion

These *in vitro *experiments quantified the spatial and temporal pressure changes during coughing in the spinal SAS with a non-communicating syrinx and/or SAS flow blockage (stenosis). The models included a syrinx and stenosis with distensible spinal column (SSED), a syrinx and stenosis with rigid spinal column (SSE), a spinal stenosis alone (SAE, no syrinx), and a syrinx with the stenosis removed (SRE) (Figure 1). Several aspects of the models were found to be important including; *influence of the stenosis, a negligible Venturi effect, influence of compliance, influence of the syrinx, LPD as a 'suck' mechanism*, and *TP for syrinx ballooning*.

While the exact etiology of SM and related disorders is not understood, some have postulated that the presence of a SAS stenosis could significantly alter the normal SAS pressure environment resulting in syrinx formation and or distension. An etiological relationship between SAS stenosis and SC syrinx is supported by typical treatment which primarily entails removal of the stenosis, such as in spinal decompression surgery for Chiari I malformation patients [43]. Pain has been hypothesized to be linked with forces acting on the SC and/or brain during coughing [1,3,8-10] which are at least partially responsible for shearing, tension, and compression of tissue. Thus, the reported difference in pressure gradients between the four *in vitro *flow models will be the focus of discussion.

### Influence of stenosis

The presence of a SAS stenosis was found to decrease spinal SAS pressure wave speed (Table 3), attenuate CSF simulated cough pulsations (Figure 3 and 4), and greatly increase LPD (Figure 5 and Table 2) during coughing. The stenosis also attenuated average SAS pressure in the direction of cough wave propagation (caudocranial). When the stenosis was removed (SRE), pressure gradients in the SAS decreased significantly. Thus, the results indicate that removal of the stenosis is a key factor needed to reduce pressure gradients in the spinal SAS during coughing. Pressure fluctuations within the syrinx cavity had little axial variation (Figure 4 and 7).

### Negligible Venturi effect

The stenosis resulted in pressure drop in the SAS but not in the syrinx which had a synergistic effect to balloon the syrinx rostral to the stenosis (Figure 4). This outward ballooning effect is reminiscent of that proposed by Greitz as a possible mechanism for syrinx expansion, in which ballooning of the syrinx was hypothesized to be the result of a Venturi effect caused by SAS flow stenosis [12,44]. However, a significant Venturi effect was not present in the current experiments with >90% SAS flow stenosis. If a Venturi effect had been present, SAS pressure caudal to the stenosis would be recuperated on the rostral side, which was not the case in SSE or SAE (Figure 4). Pressure was only slightly recuperated rostral to the stenosis in SSED. Thus, in the present experiments with >90% flow stenosis, outward ballooning of the syrinx did not require a Venturi effect, but only a decrease in SAS pressure while syrinx pressure remained uniform.

A Venturi effect was also not present in a set of similar experimental models subjected to craniocaudal CSF pulsations [37]. Interestingly, in the CSF pulsation experiments, the syrinx cavity ballooned outward at the rostral end in a similar way as in the simulated cough experiments, although these two models had the flow input direction reversed (craniocaudal for CSF pulsations and caudocranial for coughing). In the CSF pulsation experiments a 'diastolic valve effect' was found to be the underlying reason for rostral syrinx ballooning [37]. In the present coughing experiments, syrinx ballooning was a result of SAS pressure loss relative to syrinx pressure. The surprising similarities and significant differences between the coughing and CSF pulsation experiments imply that both the origin and intricacies of CSF pressure fluctuations could play an important role in syrinx pathogenesis, as a number of research studies have sought to establish [45-61].

### Influence of compliance

Increasing spinal SAS compliance, as in SSED, was found to significantly reduce peak, pulse and TPs in comparison to the rigid spinal column model (SSE) (Figures 3, 4, and 6). Reduction in these pressures will result in lowering the forces which act on the SC, which are likely correlated with damage to tissue. Increasing spinal SAS compliance in SSED was also found to decrease pressure wave propagation speed by 6-fold in comparison to SSE (Table 3). Madsen *et al*. [62] and Luciano *et al*. [63] have hypothesized that the spinal CSF may act as a mass-spring-damper system forming a notch-filter that absorbs CSF pulsations in the healthy state. If any one of the three components is altered, pathological conditions could arise.

The simulated cough data indicate that compliance of the spinal SAS, represented in Madsen's "spring", could play an important role in reducing forces which act on the SC. A number of studies have investigated changes in CSF compliance which may precede or accompany various pathologies of the craniospinal system, including SM, coining the term "pre-syringomyelia" [40,64-71]. Further development of techniques for noninvasive assessment of spinal SAS compliance such as high speed in-plane MR velocity encoding [42], axial variation in integrated CSF volume flux [72], and methods involving cerebral blood flow and CSF measurement [73,74] could be of importance.

### Influence of the syrinx

The addition of a non-communicating syrinx, in the spinal SAS with stenosis, reduced pressure forces in the SAS. This can be observed by noting the reduction in pulse and peak pressures in the SAS in SSE compared to SAE (Figure 4). The greatest pulse pressure in the SAS for all of the models was recorded in SAE to be 192 mmHg. Additionally, the syrinx decreased the wave speed and slope of pressure rise in the SAS (compare SSE and SAE in Table 3). Thus, the synergistic influence of the syrinx, when a stenosis was present, resulted in a similar influence as an increase in spinal SAS compliance. It may be possible that the presence of a syrinx, in a stenosed spinal SAS (SSE), is a mechanism for reducing forces acting on the SC in comparison to having a stenosis alone (SAE). However, removal of the stenosis, as in SRE, decreased the pressure dissociation in the SAS to a much greater extent than increasing compliance (Figure 4).

Notable differences between wave speed in the SAS and syrinx were recorded in the rigid spinal column models (SSE, SRE, and SAE), but not in the distensible spinal column model (SSED). Differences in wave propagation speed in the syrinx and SAS may be one factor influencing the potential magnitude of TPs. If the phase or the velocity of the pressure wave in the syrinx and SAS do not match, non-zero TP will occur. Martin *et al*. [36] previously cited wave speed differences in the SAS and syrinx as a possible important factor in SM progression. Bilston *et al*. investigated the importance of the relative timing of the arterial and CSF pulsations using a computational fluid dynamics model and postulated that factors which alter the arterial or CSF pulsation timing could affect fluid flow into the syrinx [32].

### Importance of pressure gradients

In order for mechanical damage to occur to neural tissue there must be pressure gradients which result in abnormal stretching, shearing, compression, and/or torsion of tissue. Thus, high peak pressure acting on a particular tissue area is not expected on its own to cause tissue damage, but rather a pressure gradient across the tissue is required. For example, high peak pressure acting on the SC in a particular region will not necessarily expand or contract the cord unless there is sufficient LPD or TP. If pressure is slowly elevated within the entire SAS, the tissue is not expected to compress because it is primarily composed of water, which is nearly incompressible. Thus, in a mechanical interpretation of the craniospinal system, pressure gradients are obligatory for tissue movement which can produce damage to tissue. In the present study, the primary pressure gradients quantified were LPD and TP.

### Longitudinal pressure dissociation as a 'suck' mechanism

LPD acted to suck fluid and tissue caudocranially in the SAS with a stenosis. In Williams' 'suck' theory for SM [6], positive deflection of the LPD trace in Figure 5, for SSE, SAE, and SSED, would indicate that a pressure gradient is available to suck fluid and/or tissue in the caudocranial direction. On the contrary, negative deflection of LPD would indicate a pressure gradient is available to suck fluid and/or tissue in the craniocaudal direction. The presence of a stenosis resulted in increasing average LPD during coughing in comparison to when the stenosis was removed (Table 2). Coughing produced the greatest peak LPD when a stenosis was present without any syrinx (SAE, Table 2, and Figure 5). Interestingly, peak LPD decreased when both a syrinx and stenosis were present (SSE). This is an indicator, in addition to the wave speed and temporal pressure results, that the syrinx may reduce forces acting on the spinal cord tissue. Note: this does not say anything about the genesis of syrinx formation, only that peak and pulse pressure forces are smaller when a syrinx and stenosis are present (SSE) than when only a stenosis is present (SAE). In fact, the average LPD increased when a syrinx was present (SSE, Table 2).

The significant level of LPD resulting from a SAS stenosis could play a role in altering fluid flow, production, and/or absorption (CSF, blood, extracellular fluid). However, significant LPD only occurred for ~ 0.1 s *in vitro*, while *in vivo *it occurred for more than 10 s [6]. In the present study, a net positive LPD was measured during coughing (Table 2, SSE and SSED) which would suck fluid in the syrinx and SAS caudocranially and act to compress vessels in the lumbar SAS (i.e. epidural venous plexus) and distend vessels in the cranium (i.e. venous sinuses). If LPD caused by a SAS stenosis is small to moderate, it is expected to be more influential on the venous system than arterial, which is at a much higher pressure. Levine hypothesized, in the presence of a SAS CSF flow blockage at the foramen magnum, a variety of activities including coughing and/or straining would result in dilation of veins caudal to the blockage and compression rostral to the blockage, resulting in mechanical stress on the spinal cord [39].

Sansur *et al*. found that peak SAS pressure during coughing decreased after decompression surgery in Chiari malformation patients with or without SM, and found higher SAS pressure in patients experiencing headache [22]. The present study indicated that peak SAS pressure increased when the stenosis was removed for experiments having a syrinx (compare SSE and SRE in Figure 4). Interestingly, peak SAS pressure was smaller rostral to the stenosis in SAE than SRE (Figure 4). However, peak LPD decreased from SSE and SAE to SRE (Figure 5 and Table 1). Given these observations, it is possible that peak LPD may be more correlated with headache and positive surgical outcome because peak pressure alone does not give information about pressure gradients that could cause damage to the tissues.

### Transmural pressure for syrinx ballooning

During a cough, the presence of a stenosis with non-communicating syrinx (SSED) would cause the syrinx to balloon outward in the further (rostral) region and become compressed in the near (caudal) region. This can be observed by the inflection of the TP traces about zero on the rostral and caudal side of the syrinx in SSE and SSED (Figure 6). When the stenosis was removed (SRE), the pressure at the far ends of the syrinx was still in opposite phase, but it oscillated quickly back and forth without producing a mean TP at either end (Figure 6). This is another indicator that removal of a spinal SAS stenosis could have a desirable influence on the system. Note: high pulse pressures alone will not necessarily damage the SC tissue. For example, SRE had the greatest pulse pressures of all the models (Figure 4), but it had small LPD and TP (Figures 5 and 6).

### Tissue damage

Overall, the synergistic effect of the LPD and TP would be to cause syrinx ballooning at the rostral end and caudocranial movement of syrinx fluid during coughing (Figures 4, 5, and 6). It is expected that TP and LPD will produce abnormal stresses on the SC, but how these stresses relate to tissue damage and symptoms such as pain is unknown. Damage to the tissue is not only a function of abnormal TP and LPD, but is likely influenced by a complex feedback and control relationship with many additional mechanical and anatomical factors including material properties, tissue geometry (location of syrinx and stenosis), SAS compliance, cranial blood flow autoregulation, and CSF transport, production, and absorption. Additionally, the anatomy of the nerve fibres and immune response may play an important role in the location and extent of tissue damage [75-78].

### Experimental assumptions and simplifications

These models were designed to mimic *in vivo *coughing conditions in a patient, but incorporated a number of simplifications. The SC and spinal column were assumed to be concentric with uniform radius along the entire spinal SAS length (Table 1). The syrinx was located concentrically within the SC (in SSE, SRE, and SSED), having a circular cross section tapered near the rostral end. Branching nerves, perfusing vessels, and spine curvature were neglected. The various meninges of neural tissue were not individually represented, but rather the spinal column and SC were assumed to each be composed of one homogenous body.

In the rigid models (SSE, SRE, and SAE), the spinal column was formed by a glass tube, and in the flexible model (SSED) it was formed by a distensible tube. The stenosis was composed of a contiguous flexible body blocking >90% of the SAS area. Additionally, the cough pulse was assumed to arise from the caudal end of the model at one location created by an abrupt pressure pulse at the flow entrance. Overall, the *in vitro *models were greatly simplified compared to *in vivo *in respect to material properties (SC and canal tissue), geometry (nerve roots, vasculature, arachnoiditis/flow blockage), and cough input. Note: while certain aspects of these experiments are applicable to patients with Chiari I malformation, namely the presence and removal of a stenosis (SAE and SRE), these models did not encompass the connection of the brain and SC and therefore no piston action of the lower cerebellar tonsils was present. Future *in vitro *investigations could include the craniospinal junction to investigate the pressure environment in Chiari I malformation.

### Comparison of in vitro results to in vivo measurements

The simulated cough pressures can be compared to *in vivo *measurements in terms of temporal pressure maximum and minimum, slope of the pressure rise, and LPD. While maximum and minimum pressures in the rigid models were high, they are within the bounds of clinically measured SAS pressures during coughing [3,8,9,19,40,41]. Williams *et al*. measured peak pressure during coughing to be ~ 80 mmHg [6] and Sansur *et al*. measured it to be 125 mmHg [22]. Peak pressures in the rigid models were similar in magnitude although smaller in the flexible model (Figure 4). The maximum slope of the SAS pressure rise in the rigid model (SSE, SAE, SRE) varied from ~ 8,000 to 13,200 mmHg/s (Table 3). Maximum pressure rise slope in the distensible model (SSED) was ~ 1,700 mmHg/s. Sansur *et al*. measured SAS pressure rise during coughing to be 170 and 210 mmHg/s in patients and healthy volunteers, respectively [22]. SAS pressure rise during coughing was also quantified by Williams [1].

The steep slope of the *in vitro *pressure rise is likely due to the cough pulse being more abrupt than *in vivo *and because the rigid models were less distensible than *in vivo*. However, in the case of SSED, wave speed in the SAS was 24 m/s, which is similar in magnitude to that measured by Jackson and Williams *in vivo *of 13.5 m/s [79], but faster than estimated by Greitz of 4 m/s using MR velocity [44]. Carpenter asserted that Williams' estimated wave speed was likely to be faster than actually occurring *in vivo *and that his measurement should be 4 to 5 m/s [25]. Wave speed in the rigid models (SSE, SRE, and SAE) was much faster than measured *in vivo *(Table 3). A summary of wave speed measurements in the spinal SAS was given by Kalata *et al*. [42].

Williams *et al*. presented *in vivo *LPD measurements on 37 SM patients with hindbrain abnormality and found that 24 of them had a "valvular action...allowing fluid to move upwards easily but only with more difficulty and delay...move downward again." [6]. The valve action worked to transmit LPD pressure quickly during the onset of coughing or valsalva (<1 s), but slowly afterwards (>10 s). Williams' recorded a maximum LPD during coughing onset to be 100 mmHg (P_lumbar _- P_cervical_). LPD can be estimated from intraoperative jugular compression results for patients with SM and Chiari I malformation reported by Heiss *et al*. (Queckenstedt's test) to be as large as -20 mmHg (P_lumbar _- P_ICP_) [40]. The Queckenstedt's test has effectively the opposite influence on the spinal SAS as coughing [6], hence the opposite sign of LPD in Heiss' results in comparison to Williams'. In SSE and SSED, significant LPD only persisted between the lumbar and cervical SAS for 0.1 s after the cough input, returning quickly to LPD equilibrium (Figure 5). The delayed return of LPD in the spinal SAS in Williams' study may be because the *in vitro *models did not incorporate the complex vascular network in the SAS, which adds additional communication with the intra-abdominal, venous, intracranial, and arterial pressure [35,39]. Additionally, the *in vitro *models lacked any hindbrain herniation causing valvular action, albeit they may have had a modest valve action between the syrinx and stenosis [37].

While the *in vitro *experiments differed from *in vivo *by neglecting to incorporate blood vessels and piston action of the brain, the peak *in vitro *LPD measurements (Table 2) in SSE and SSED were within the range recorded by Williams [6], and Heiss [40]. Also, SSE and SSED had a positive difference between the lumbar and cisternal pressure (P_lumbar _- P_syrinx_) after coughing (Figures 3 and 4), which was similar in sign and magnitude to that recorded by Williams [6]. The amplitude of a cough pressure pulse in healthy subjects was recorded by Williams to attenuate by ~ 80% from the lumbar to the ventricular SAS [3]. In SRE, SAE, and SSED the pressure pulse amplitude attenuated by 91, 58, and 29%, respectively (attenuation is the difference in pulse pressure measured between 4 and 32 cm in the SAS in Figure 4). However, in SSE the pressure pulse amplitude increased by 20%.

The cited literature and the present study suggest that the SAS pressure environment during coughing is sensitive to the complex interaction of the SC, syrinx, and stenosis. For this reason it is challenging to compare directly the *in vivo *and *in vitro *data. However, given the careful consideration of the biomechanical factors of the *in vitro *experiments (e.g. geometric, flow, pressure, and material properties), the general trends observed *in vitro *are likely representative of the *in vivo *pressure environment. These pressure measurements are useful as they describe the individual influence of biomechanical factors in the spinal SAS, which may help better understand craniospinal disorders. Additionally, the measurements provide data that is difficult to acquire *in vivo *for comparison to numerical models [23-29,31,80,81].

## Conclusions

This study examined the highly complex pressure environment in the spinal SAS with a stenosis and syrinx under simulated coughing conditions using *in vitro *models. Alterations in geometry, compliance, and presence of the syrinx and stenosis had significant impact on the wave speed and pressure gradients (TP and LPD). The presence of a >90% SAS stenosis resulted in a caudocranial pressure drop in the SAS but not in the syrinx and was found to balloon the syrinx outward at its rostral end and be compressed at the caudal end. The LPD caused by a spinal SAS stenosis acted to suck fluid and tissue caudocranially. Pressure forces in the spinal SAS were reduced by the presence of a syrinx in the SC and also by an increase in spinal column compliance. Pressure fluctuations within the syrinx cavity had little axial variation. Overall, the *in vitro *results support the hypothesis that the removal of a spinal SAS stenosis is a key factor needed to reduce CSF pressure gradients produced during coughing.

## Abbreviations

CSF: cerebrospinal fluid; LPD: longitudinal pressure dissociation; SAE: stenosis alone model (no syrinx present); SAS: subarachnoid space; SC: spinal cord; SM: syringomyelia; SRE: stenosis removed model (with syrinx); SSE: stenosis and syrinx model; SSED: stenosis and syrinx model distensible; TP: transmural pressure.

## Competing interests

The authors declare that they have no competing interests.

## Authors' contributions

BM carried out the experiments and was the primary contributor to text. FL assisted in conducting experiments and revision of the text. Both authors have read and approved the final version of the manuscript

## Supplementary Material

Additional file 1 ***In vitro *cerebrospinal fluid pressure measurements recorded during the coughing experiments**. A Microsoft excel file (data_coughing.xls) contains the relative pressure (in mmHg) recorded in each of the experimental models (SSED, SSE, SAE, and SRE) at distinct axial coordinates (4, 8, 12, 16, 20, 24, 28, and 32 cm) within the spinal subarachnoid space and syrinx (when present). A summary of geometric and material properties for each model is indicated in Table 1. Location of the flow input, sensors, syrinx, and stenosis is indicated in Figure 1. Pressure data was recorded by each sensor during coughing at 16 bits 17 kHz for a total of 4000 samples. Pressure measurements are provided relative to the initial pressure at each sensor before the cough was produced (details in text). Click here for file
